# From the archives: Targeting proteins to plasmodesmata, germination in the desert, and functional diversity of KNOX proteins

**DOI:** 10.1093/plcell/koae148

**Published:** 2024-05-14

**Authors:** Vicky Howe

**Affiliations:** Assistant Features Editor, The Plant Cell, American Society of Plant Biologists; Department of Developmental Genetics, Heinrich-Heine University, Düsseldorf 40225, Germany

## August 2023: Unusual plasmodesmal targeting signals

Plants control the cell-to-cell movement of many signaling molecules by adjusting the permeability of plasmodesmata, membrane-lined channels connecting adjacent cells. One family of proteins that regulate plasmodesmal permeability is the aptly named PLASMODESMATA-LOCATED PROTEINS (PDLPs). Despite being plasma membrane associated, PDLPs do not move freely throughout the cell periphery. Rather, they are retained specifically at the plasma membrane lining plasmodesmata. How this is achieved has been something of an enigma for researchers, as no sequence-specific plasmodesmal-targeting signals have been identified. Recently, Robles Luna et al. (2023) developed a new machine-learning algorithm to predict plasmodesmal-targeting sequences in PDLPs. Although mutating or deleting these sequences reduced the plasmodesmal localization of the PDLPs, the targeting signals differed in length (5–14 amino acids), number (1 or 2), and sequence between the 8 PDLP members. The only real commonality was their position, encompassing ∼30 amino acids proximal to the transmembrane domain, termed the JMe (for juxta-membrane, extracellular). Despite a lack of sequence or structural similarities, deleting the equivalent JMe in 4 unrelated plasmodesmata-localized proteins also perturbed their plasmodesmal localization. Conversely, swapping their JMe with the PDLP5 JMe, or vice versa, retained PD localization. The authors therefore concluded that these were location-specific, rather than structure- or sequence-specific, plasmodesmal-targeting signals, and that they may be a common feature of plasmodesmal transmembrane proteins. The group speculated that plasmodesmal proteins may utilize specialized transport machinery that recognizes each unique JMe and delivers the proteins to the plasmodesmata. Determining what these transport components are, and the mechanisms that facilitate plasmodesmal targeting, are next on their to-do list.

## August 2019: Emergent protective organogenesis in date palm

**Figure 1.**
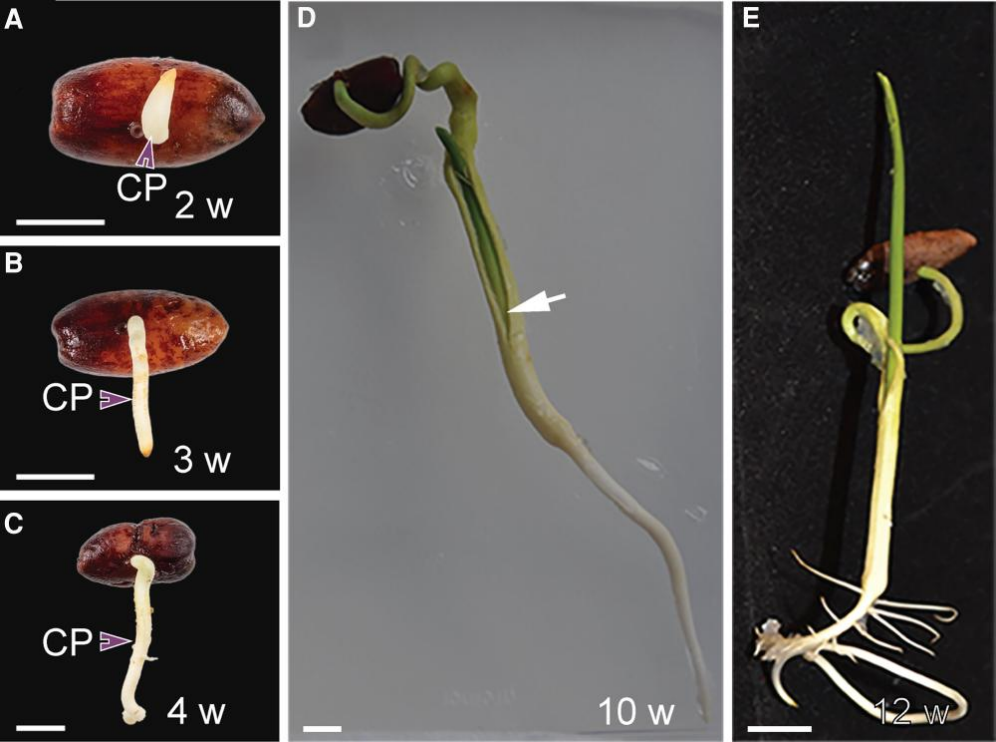
Remote germination protects developing date embryos. **A to C)** Following germination, the embryo-containing cotyledonary petiole (CP, purple arrowheads) emerges and grows toward the soil. A) In the early stages, embryonic development is paused. B, C) After 3 weeks, development resumes within the CP, until (**D**) the true leaves emerge 10 weeks after germination. **E)** The CP tip forms the primary root and the seedling emerges from the soil 12 weeks after germination. Scale bar = 1 cm. Adapted from Xiao et al. (2019), Figure 1.

Next, we delve into desert life. In more hospitable environments, seedlings start generating organs immediately after germination, emerging from the seed coat with a recognizable shoot and root. Such an approach in the desert, however, would expose delicate meristematic tissue to damaging elements. Five years ago, Xiao et al. (2019) uncovered a multi-faceted strategy adopted by the date palm (*Phoenix dactylifera*) to protect the developing embryo and prepare the future seedling for a harsh desert existence. First, the developing embryo is physically protected by being encased in a tuber-like structure, the cotyledonary petiole, that emerges from the seed and grows into the earth in a process called remote germination (see Fig. 1). Additionally, while the cotyledonary petiole is exposed to the dry, hot soil surface, embryonic development is paused, providing a molecular level of protection until the embryo is safely submerged. Development then continues within the cotyledonary petiole until the developed seedling finally emerges around three months after germination (Fig. 1E), already equipped with true leaves and the beginnings of a specialized root system adapted to drought, salinity, and low nutrient availability. Furthermore, the group found that genes involved in bacterial association were highly expressed in the roots. This corroborates growing evidence that bacterial symbioses facilitate nutrient uptake and salinity tolerance in desert plants (Cherif et al. 2015; Shamim et al. 2022), which is an exciting new avenue being explored in agriculture (Zhang et al. 2020).

## August 1999: KNOX domain functional specificity in tobacco

Finally, after the first plant homeobox gene, *KNOTTED1*, was cloned from maize in 1989 (Hake et al. 1989), related genes were identified in numerous model plant species. Kerstetter et al. (1994) coined the term KNOX (for KNOTTED1-LIKE HOMEOBOX) to describe this class of transcription factors, which were quickly being recognized as key regulators of diverse developmental processes. In 1999, Tomoaki Sakamoto and colleagues (Sakamoto et al. 1999) conducted the first systematic functional analysis of conserved KNOX protein domains in transgenic tobacco (*Nicotiana tabacum*). The group observed that overexpression of 3 tobacco KNOX proteins caused different degrees of phenotypic severity. Using domain-swapping experiments, Sakamoto and coauthors showed that phenotypic severity was the product of both N- and C-terminal sequences. However, they speculated that structural variation of the second KNOX domain likely specified interaction with different accessory proteins. Indeed, recent studies show that multiple sites in the KNOX1 and KNOX2 domains are particularly prone to mutations that affect protein structure and function (Meng et al. 2020). Subsequent research has also identified a multitude of KNOX interaction partners, accounting for their distinct functions through different signaling pathways (see Jia et al. 2023 for a comprehensive review). As many of the pathways regulated by KNOX proteins affect agriculturally relevant traits, they are seen as an attractive target for crop improvement.

## Dive Curated Terms

The following phenotypic, genotypic, and functional terms are of significance to the work described in this paper:

PDLP5 Gramene: AT1G70690
PDLP5 Araport: AT1G70690
